# The significance of occupations, family responsibilities, and gender for working from home: Lessons from COVID-19

**DOI:** 10.1371/journal.pone.0266393

**Published:** 2022-06-13

**Authors:** Lara Minkus, Nicolai Groepler, Sonja Drobnič

**Affiliations:** 1 SOCIUM Research Center on Inequality and Social Policy, University of Bremen, Bremen, Germany; 2 Flensburg University, Flensburg, Germany; FAME|GRAPE, POLAND

## Abstract

Before the pandemic, many employers were hesitant to offer their employees the option of working from home. However, remote working has been widely adopted during the pandemic as one of the key methods of controlling the spread of the virus. The measure encountered a widespread acceptance and it is likely that the demand for work from home as a flexible work arrangement will persist also after the pandemic has ended. Although numerous studies have addressed the role of remote work during this crisis, as of yet we lack thorough research jointly addressing the question on how occupations/job characteristics on the one hand and family/household responsibilities on the other are associated with the propensity of working from home, and how gender cuts across those aspects. Using the COVID-19 survey of the German Family Panel (*pairfam*), covering the peak of the first wave of the pandemic in 2020, together with information from *pairfam* panel waves conducted before the pandemic, as well as a special evaluation of the 2019 German Labor Force Survey, we are able to address this gap. Employing linear probability models on a sample of 1,414 men (N = 641) and women (N = 773), our results show that occupational traits, especially the gender composition of an occupation, are an important predictor for working from home. Women employed in female-dominated occupations are less often in a position to work from home. Furthermore, our study confirms that it is particularly the highly educated, as well as those who work in high-prestige occupations, who are able to work from home. Family configurations and care obligations are less influential upon the transition to homeworking, even in times of an unprecedented situation of school and daycare closures and increased parental responsibilities for children’s (early) education.

## Introduction

As the current COVID-19 pandemic forces reconsideration of our accepted work patterns, working from home is certainly one of the issues that have drawn the most attention. In many countries, remote working was widely adopted as a solution to the challenges posed by social distancing, such as avoiding contact in the work place and using public transportation. Unlike many other measures, work from home (WFH) received near-universal praise in the media. Indeed, studies have shown that not working from home is a significant risk factor for contracting a COVID-19 infection [1]. Empirical evidence suggests that a one percentage point increase in WFH would reduce the infection rate in Germany by as much as eight percent [2].

Even though many employers were previously hesitant to allow their employees more flexibility in organizing their working day, the pandemic–acting as an external shock to the system–has forced a rapid revision of this practice. Moreover, the experience during the pandemic triggered political debates on whether employees in general should have the legal right to work from home if it is reasonably practicable to do so. Therefore, it is likely that WFH will not disappear from the political agenda and as a desired job option for some employees when the pandemic will end. It is thus important to understand who has the chance to work from home and which potential factors are associated with WFH. Is remote working a privilege of specific occupational groups or can it spread as a flexible work arrangement and benefit employees most in need of flexible working?

Tentative evidence from prior to the pandemic suggests that the highly educated and top earners [3, 4], as well as incumbents of certain occupations [3, 5] were more likely to be granted the right to work from home, while family and care responsibilities were not decisive for WFH [3]. During the pandemic, the necessity of working from home arose alongside the closure of daycare facilities and schools, thus requiring parents to take responsibility for childcare and education at home. Only parents in the so-called essential occupations, such as health services, were entitled to emergency childcare. As other options were heavily restricted, such as relying on grandparents and other informal support networks not living in the household, parents alone were, in most cases, responsible for taking care of their pre-school and school-aged children. Besides various leave arrangements provided by employers and state programs—which are comparatively short-term solutions with restrictive conditions—working from home seems a suitable answer to the problem of childcare under such circumstances. Remote working is certainly not possible for all employees, but it can be assumed that parents with care responsibilities are particularly likely to make use of this option. Working from home can provide flexibility in reconciling paid work and care, although the challenges, particularly during periods of school and daycare closure, should not be underestimated.

WFH under the circumstances of COVID-19 can thus be understood as a test of the *potential* for working from home under general conditions, as the pandemic revealed the feasibility ceiling for this form of flexible work arrangements. Therefore, it is due time to re-evaluate and juxtapose the importance of occupations/job characteristics on the one hand and family/household arrangements on the other for the propensity of working from home. Do education and occupational status continue to drive WFH or have employers responded to the unprecedented need for care work by granting remote work also to those with increased care responsibilities? Gender as a cross-cutting dimension is of particular interest, given that women are the main caregivers but also tend to work in different occupations than men. Differences in WFH between women and men can thus occur because of occupational segregation by gender in the labor market or because women and men differ in their domestic responsibilities and presumably seek WFH differentially.

Using data from the German Family Panel (*pairfam*), particularly its additional web-based COVID-19 survey, which focused on *pairfam* respondents’ family situations during Germany’s first wave of the coronavirus pandemic, as well as a special evaluation of the 2019 German Labor Force Survey (LFS), this study seeks to examine which employment-related characteristics and family responsibilities are primarily associated with individuals’ working from home during the pandemic. We also address gender considerations to disentangle the critical interplay between gender, occupational structure, industry/sectoral composition, and household constellations.

### Working from home: Occupations, care work, and gender before and during the pandemic

Previous research suggests there are considerable disparities in the distribution of both flexible work schedules and flexible work locations among demographic and job sub-groups. In particular, the ability to work from home is an option primarily available to highly educated, high-status employees [6–8], and is commonly not available to relatively lesser-educated workers. Overall, in the EU, the share of employees who *usually* work from home remains very low, and some EU countries do not provide data on this phenomenon [9]. As far as gender differences are concerned, findings are inconsistent. Available research indicates that methodological differences and country constellations lead to contradictory conclusions regarding the relative magnitude of gender-specific differences. When working from home is defined as spending at least half of the working time at home, and taking into account only employees, Plantenga and Remery [9] find that in most European countries women are somewhat more likely than men to work from home. This is in line with findings from the USA [7]. However, if various constellations of working from home are considered, as well as the self-employed, the results are reversed: data for Germany show that men are more often in a position to work from home than women [3, 10, 11]. Similarly, for the US it was found that married men have most access to telecommuting [12].

Why should gender differences in this respect matter? Flexible daily work scheduling and the location of work may assist employees to better manage their working and family life, especially at times when family responsibilities pose a significant demand. Flexible work arrangements may be particularly beneficial and desirable for women who still do most of the housework and care, while men remain the primary breadwinners [13–16]. However, based on data from the German Socioeconomic Panel (SOEP), Brenke [5] presents evidence that family obligations do not play a key role in predicting who works from home, i.e., single people without children work from home as often as lone parents. Based on the American Time Use Survey 2017–2018, Alon et al. (2020) find evidence that single parents are proportionally less often able to telecommute compared to married men and women with children [12]. Again, methodological differences and the composition of the samples are likely to lead to discrepancies in findings. A study based on the German Labor Force Survey (*Mikrozensus)* suggests that, prior to the pandemic, parents in Germany did work from home slightly more often compared to workers without children, and amongst parents, fathers more often than mothers [3]. For the US it was found that fathers have the most access to remote work, while mothers made use of the option to WFH most often once they were offered to work remotely [12, 17].

Previous research has shown that women do not work from home for two main reasons. First, some occupations are more suitable for such working arrangements than others [12, 18–21]. For Germany, Brenke [5] points out that a “Home Office”—or simply homeoffice, as it is becoming commonly termed in Germany—is particularly common in the banking and insurance sectors, as well as the public sector. In contrast, the female-dominated hospitality and health sectors, as well as the male-dominated manufacturing sector, offer comparatively few opportunities to work from home. Second, whether telework is an option at the workplace is significantly dependent upon the extent to which the ideal worker norm is upheld in a particular workplace [8]. The ideal worker norm that prevails in “gendered organizations” [22] establishes a workplace philosophy that paid work is the only responsibility of employees who are wholly committed to their jobs. Thus, a decreased physical presence at the workplace can carry negative consequences. Indeed, evidence suggests that workers who request flexible work arrangements are evaluated more negatively than those who do not [23]. These consequences are feared by employees who consider working from home [24, 25], and women are more often concerned than men about the negative impact of telework on their careers [8].

Comparative research shows that, prior to the pandemic, Germany’s rates of remote work were below the European average [3]. Although it is estimated that the contemporary German labor market offers the potential for almost 40% of all workers to work from home, prior to the pandemic only 12% did so, at least partially [5]. The current pandemic has witnessed a dramatic increase in working from home [4, 26, 27]. As a precaution to prevent the further spread of the virus by reducing social contacts, the government imposed lockdowns—including the closure of non-essential businesses—and urged employers and employees to perform work from home where feasible.

In addition to the increase in remote working, lockdown regulations in Germany in Spring 2020 also required that schools and daycare facilities close during the first peak of the pandemic. This obviously led to an immense increase in care responsibilities for families. Parents were expected to assume control of childcare and support the educational needs of their children. Accordingly, the time spent on childcare rose sharply [28]. Although men increased their share of domestic and care work during this time, in response to changes in working hours [3, 29], women continued to carry out the vast majority of housework and caring duties [28, 30–32]. Studies benefiting from monthly panel data in Germany and the UK demonstrate that increased participation of male partners in housework or childcare tended to be small at most and only temporary [33–35]. Overall, this means that the division of domestic labor remains at least as unequal as it was prior to the pandemic. This striking degree of stability in the aggregate does not mean that there may not have been more pronounced changes for certain subgroups of couples: The increased contributions of male partners seem to be driven mainly by constellations with mothers with a strong labor market attachment without the option to work from home [33, 36]. Another German study detected some shift toward the extreme end of the distribution where mothers take over childcare and housework almost completely [37]. However, there are some inconsistencies between studies in some of the more nuanced findings, which may well be due to differences in samples and model specifications. WFH is thus all the more crucial for women during the pandemic. Recent research suggests that remote work helped mothers to maintain their level of paid work hours to a greater extent than mothers working on-site, who more often withdrew or reduced work time for family-related reasons [38]. Nevertheless, descriptive evidence suggests that it was not employees with families that were given the opportunity to work from home in the first place. Rather, it seems that it was particularly highly educated workers, and workers with high incomes, who were able to take advantage of the opportunity to work from home [4, 39], a continuation of the trend established before the pandemic [3, 5, 38].

During the course of the pandemic, societies have learned that their operation heavily depends on so-called “front-line workers”. These essential workers, be they health professionals or workers at grocery stores, are predominantly female [40, 41]. Furthermore, while about half of all women with children work in an essential occupation, the respective share of fathers amounts to only a third [4]. Most of these essential workers cannot work from home [26, 42]. Thus, the occupation-related reasons that are associated with the likelihood of whether an individual is able to work from home or not have most likely gained importance during the current health crisis.

Whether women can work from home or are obliged to work from their employer’s location not only bears potential consequences for work–family conflicts, but also has severe consequences for women’s health during the pandemic. Women work in occupations where personal contact is crucial, which, in turn, results in the increased possibility of exposure to infection in the workplace [18]. Lewandowski et al. [41] show that the gender gap in exposure to the disadvantage of women can be largely attributed to patterns of sectoral segregation. These results indicate the importance of the gendered structure of contemporary labor markets, which carry different implications for men’s and women’s health.

At first sight, the available evidence on differences between women and men working from home during the pandemic appears inconsistent. In particular, results are difficult to compare due to methodological variability. Several studies indicate little difference in the rise of telework between women and men in Germany compared to prior to the pandemic [32, 42–44]. Reflecting comparable developments in the female and male labor force, descriptive evidence suggests that, in absolute numbers, the share of men working from home during the pandemic remains somewhat larger compared to women [4, 45, 46]. However, for a specific group of German employees—those in private companies with more than 50 employees who use digital information and communication technology in their work—Frodermann et al. [47] find a higher share of the transition to telework among female employees than male. Likewise, Bick et al. [19] and Reichelt et al. [44] identify a larger rise in working from home among women than men in the United States, indicating the potentially crucial role of the structure of the labor market. However, a systematic assessment of how the pandemic reveals the feasibility for WFH considering the role of occupational traits, family responsibilities, and personal characteristics for men and women has yet to be performed.

## Data and methods

### Data

This study uses data from the German Family Panel (*pairfam*) [48]. Starting in 2008, *pairfam* provides data on the formation and development of intimate relationships and families in Germany [49]. Data collection is consistent with the ethical standards for the treatment of human subjects (German Research Foundation, Reference Number 19016KH). Informed consent was obtained verbally from all participants included in the study. Data are collected annually from a nationwide, random sample of, initially, three birth cohorts (1971–73, 1981–83, and 1991–93). In Wave 11, the sample was enlarged by the addition of a cohort of younger respondents, born 2001–2003. A special feature of the panel is its multi-actor design: in addition to the anchor respondents, also their partner, child(ren) and parents are interviewed.

To assess and monitor the life circumstances and consequences that accompanied the COVID-19 pandemic, *pairfam* implemented an additional online survey, administered from mid-May to mid-July 2020 [50–52]. Hence, the *pairfam* COVID-19 Survey concerns the time shortly after the first strict period of lockdown in Germany. To asses previous working arrangements, job characteristics and demographics, we also used *pairfam* waves ten to twelve. Additionally, we used information on the gender composition of occupations, which is based on data from the 2019 German Labor Force Survey (*Mikrozensus*) and provided by the German Federal Statistical Office [53], the latter data can be found in S1 Dataset.

Starting with 3,018 interviewees of the supplementary *pairfam* COVID-19 survey, we restricted our sample to respondents who reported being employed or self-employed, irrespective of the type of employment contract or other concurrent activities stated, e.g., schooling or training. After this initial loss of 1,057 observations, we excluded respondents who additionally to their employment status reported having been laid off or being on a special paid or unpaid leave due to the COVID Pandemic, which makes a loss of another 191 observations. After deleting observations with missing information regarding the variables of interest (N = 356), the sample includes 1,414 respondents between 16 and 49 years of age.

### Variables

The dependent variable is whether the respondent (partially) worked from home at the time of the survey. More specifically, respondents were asked whether their working arrangements changed because of the COVID-19 pandemic. The binary dependent variable was set to one if respondents reported working entirely or partially from home. We also included respondents who had worked from home already before the pandemic and reported that nothing had changed in their working arrangements (N = 55).

In order to account for **family demands** that resulted from the pandemic, we differentiate between childcare and housework. For childcare, we created three dummies to distinguish whether, first, care is provided completely/predominantly by the respondent, second, it is evenly distributed or completely/predominantly done by the partner or provided by someone else, or, third, there are no children in the household. Likewise, dummies for housework distinguish whether household chores are done completely/predominantly by the respondent, they are evenly distributed or completely/predominantly done by the partner or carried out by someone else, or the respondent does not live together with a partner.

To test the relationship between **occupational attributes** and working from home, we employed several occupation-specific independent variables. First, we created three variables measuring gendered occupational segregation. Information on occupational segregation was generated by calculating the percentages of women in each occupation of the 2010 three-digit job classification, using data from a special evaluation of the 2019 German Labor Force Survey, which was provided by the German Federal Statistical Office [53]. We then coded the variable into three categories: “female-dominated” if an occupation had 70 percent or more women, “mixed occupations” if the share of women in an occupation was between 30 and 69 percent, and male-dominated occupations if less than 30 percent of incumbents were women. This information was then merged with the 2010 three-digit job classification variable in the core *pairfam* dataset. It is important to note that the job classification is not included in the supplemental *pairfam* COVID-19 data; therefore, we had to retrieve this information from adjacent survey waves. Secondly, we measured occupational prestige by including the International Socio-Economic Index of Occupational Status (ISEI) [54]; note that this information has also been extrapolated from adjacent *pairfam* waves. We divided the index by 10 so as to be able to interpret the coefficient as an increase of 10 ISEI points.

Further, we included information on **educational level**, using a categorical variable based on the CASMIN (Comparative Analysis of Social Mobility in Industrial Nations) classification scheme retrieved from adjacent waves of *pairfam*: lower secondary education and below (“low education”), higher education entrance qualification and below (“intermediate education”), tertiary education (“high education”), and a residual category comprising those who are not only employed but also “currently enrolled” in the educational system. This information, too, needed to be retrieved from adjacent *pairfam* waves.

We controlled for birth cohort and whether the lock-down regulations were still in place at the time of the interview (= 1). We further account for the composition of the sample by controlling for region (eastern vs. western parts of Germany), migration background, and settlement structure (rural vs. urban), variables that were also retrieved from adjacent waves. Please note that we made a deliberate decision against weighting in our analysis, and instead included aforementioned relevant sample composition variables directly into the models.

### Method

We first conducted a bivariate analysis on working from home (‘homeoffice’) and its demographics. We then explored the association between working from home and our independent variables using multivariate linear probability models (LPM) [42]. LPMs have many advantages over conventionally employed models for binary variables such as logistic regressions, for example that coefficients in LPM can be meaningfully compared across models [55]. Formally this model can be described as:

$$P(Y=1|x)=\beta_{0}+\beta_{1}x_{1}+\beta_{2}x_{2}+\cdots +\beta_{k}x_{k}$$

Since linear probability models are known to suffer from heteroskedasticity, we applied robust standard errors [56]. Analyses were conducted using Stata 16.1. Replication materials can be found in the supporting information section of this article (S1 Materials).

## Findings

### Descriptive findings

Descriptive statistics for the employed respondents who completely or partly worked from home during the pandemic and those who did not are displayed in Table 1. T-tests compare the differences between the two groups. Covariates vary largely with regard to occupational characteristics and education. Highly educated respondents and those who work in higher prestige occupations significantly more often worked from home, as did respondents working in an occupation with a mixed gender composition. This is in line with previous findings [4] and underscores that it is especially those with high education who worked from home during the first wave of the pandemic. With respect to family responsibilities, we find almost no significant descriptive differences between the two groups.

**Table 1:** Description of the sample by working from home (WFH) and the differences between the two groups.

	WFH		No WFH		Δ
	Mean	SD	Mean	SD	
Women (= 1)	0.53	(0.50)	0.56	(0.50)	-0.03
*Family attributes*					
Care: completely/mostly me	0.16	(0.37)	0.15	(0.35)	0.02
Care: even/mostly partner/other	0.32	(0.47)	0.31	(0.46)	0.01
Care: no children	0.52	(0.50)	0.55	(0.50)	-0.03
Chore: completely/mostly me	0.28	(0.45)	0.29	(0.45)	-0.01
Chore: even/mostly partner/other	0.49	(0.50)	0.45	(0.50)	0.05^+^
Chore: single/non-cohabiting	0.22	(0.42)	0.26	(0.44)	-0.04^+^
*Occupational characteristics*					
Men‘s occupation	0.26	(0.44)	0.25	(0.43)	0.01
Mixed occupation	0.49	(0.50)	0.38	(0.49)	0.11***
Women’s occupation	0.25	(0.43)	0.37	(0.48)	-0.12***
ISEI/10	6.59	(1.55)	4.76	(1.88)	1.83***
*Education*					
Low education	0.02	(0.15)	0.09	(0.28)	-0.06***
Intermediate education	0.27	(0.44)	0.61	(0.49)	-0.34***
High education	0.68	(0.47)	0.25	(0.43)	0.43***
Currently enrolled	0.03	(0.17)	0.05	(0.21)	-0.02^+^
*Controls*					
Lock-down in place (= 1)	0.91	(0.29)	0.83	(0.37)	0.08***
East (= 1)	0.18	(0.38)	0.25	(0.44)	-0.08***
Migration background (= 1)	0.12	(0.32)	0.15	(0.36)	-0.03^+^
Rural (= 1)	0.16	(0.36)	0.29	(0.45)	-0.13***
Cohort 1971–1973	0.30	(0.46)	0.25	(0.43)	0.05*
Cohort 1981–1983	0.44	(0.50)	0.39	(0.49)	0.04^+^
Cohort 1991–1993	0.25	(0.43)	0.31	(0.46)	-0.06*
Cohort 2001–2003	0.01	(0.11)	0.05	(0.21)	-0.03***
Observations	734		680		1414

Notes: Based on *pairfam*-COVID-19 survey and *pairfam*, release 12.0, and a special evaluation of the German LFS 2019, own calculations, not weighted; SD = standard deviation in parentheses. Asterisks indicate the significance of differences in means

^+^ *p* < 0.10

* *p* < 0.05

** *p* < 0.01

*** *p* < 0.001.

**Fig 1:**
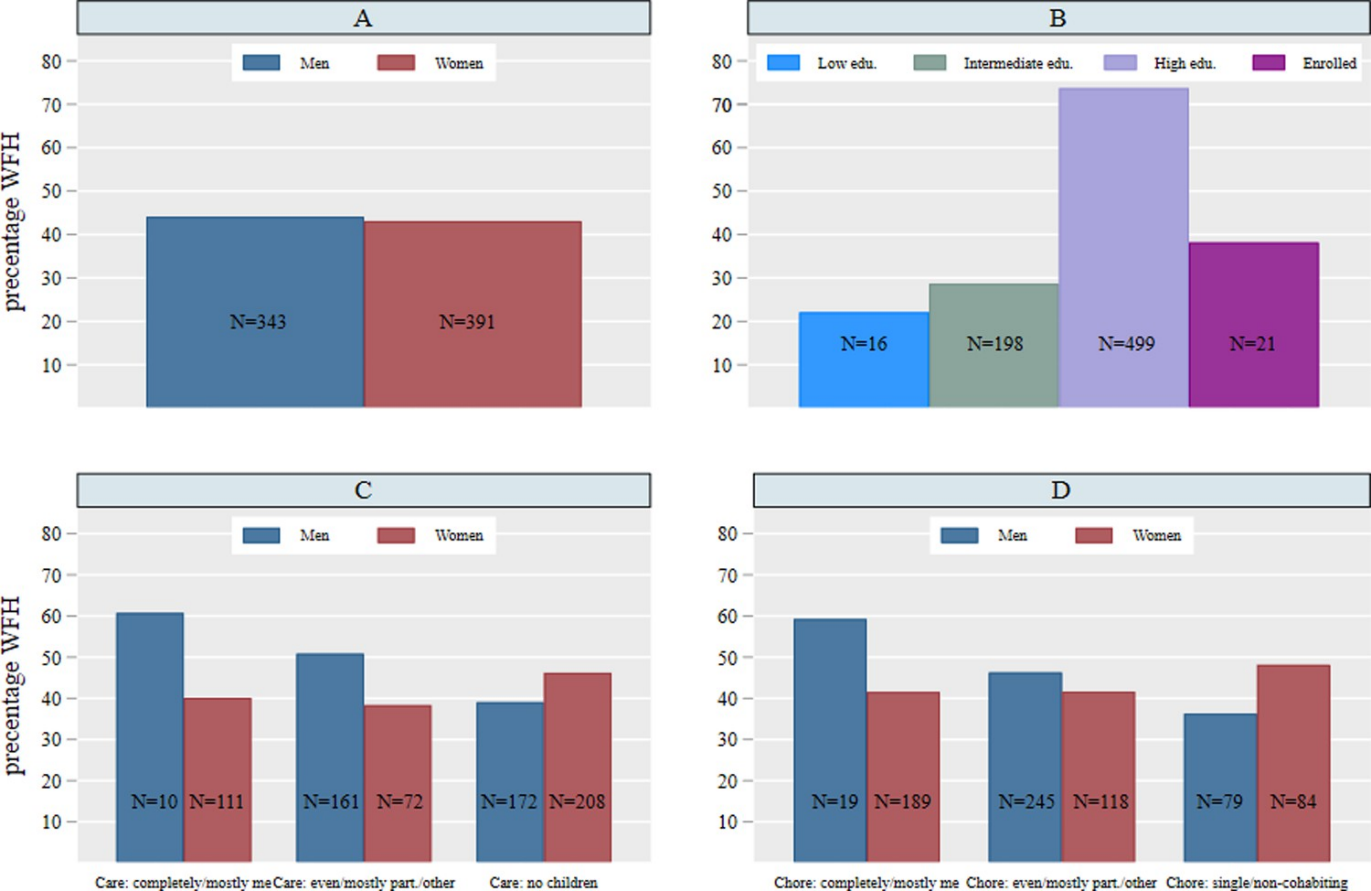
Work from home by gender (A), educational level (B), care (C) and household chores (D). Note: Based on *pairfam*-COVID-19 survey and *pairfam* Release 12.0, own calculations, weighted.

Weighted percentages (not shown) indicate that among the working population in Germany, approximately 44% worked from home during the first wave of the pandemic. Interestingly, this is close to the pre-pandemic estimates that the contemporary German labor market offers the potential for about 40% of all workers to work from home [5]. Graph A in Fig 1 reveals that the weighted gender difference in working from home is basically non-existent. Graph B further illustrates the profound gap between educational levels in the prevalence of working from home (weighted). As seen in Graph B, 75% of highly educated respondents reported working from home at least partially, while only 21% of the low education group reported doing so.

Regarding family demands (Graph C and D in Fig 1), the descriptive evidence presented suggests that the association between having the main responsibility for childcare and housework and WFH is stronger for men than for women; however, the sample sizes are very small. Thus, in rare households in which men are sole or primary carers and/or housemakers, men are predominantly working from home. This association is less pronounced in families, in which childcare and housework are evenly split or the partner does most or all of the work. Women are more likely to work from home if they are single or do not live together with children. To test more thoroughly whether this descriptive evidence holds, we now turn to the linear probability models.

### Multivariate analysis

**Fig 2:**
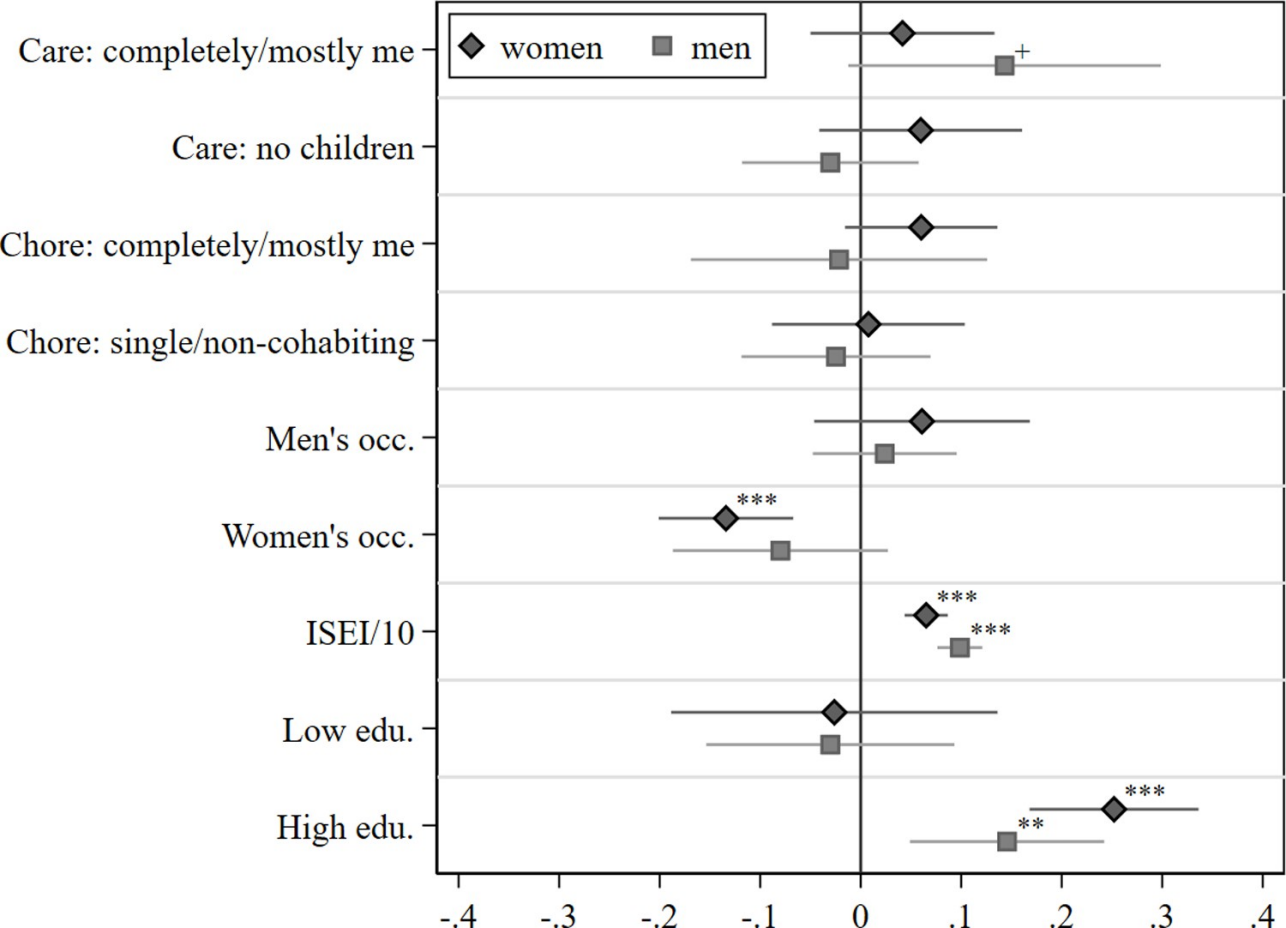
Work from home regressed on family responsibilities, occupational characteristics, and education. Note: Plot of Linear Probability Model coefficients (see S2 Table for the full models). Based on *pairfam*-COVID-19 survey and *pairfam*, release 12.0, and a special evaluation of the German LFS 2019, own calculations, not weighted. Reference groups: Care: split even/mostly partner/completely partner; Chore: split even/mostly partner/completely partner; mixed occupation; intermediate education. Controls (not shown): enrolled in education, East, migration background, rural, cohorts. ^+^ *p* < 0.10 * *p* < 0.05, ** *p* < 0.01, *** *p* < 0.001.

We regressed working from home on household and occupational characteristics, educational level, and control variables that are assumed to be associated with working arrangements during the pandemic. The estimated coefficients based on the linear probability models for the whole sample, and separately for men and women, are presented in S2 Table and illustrated in Fig 2. As evident in Fig 2, only a few of our covariates are significantly associated with the likelihood of working from home. In addition, there is only one significant difference in covariates between men and women (see also S2 Table).

Considering the results in detail, we find that *occupational characteristics* are an influential predictor for WFH. Working in a female-dominated occupation is negatively associated with the overall likelihood of working from home by about 13 percentage points (see S2 Table). Further examination of this association reveals that it is particularly women in female-dominated occupations who were less likely to work from home during the first peak of the pandemic, while the coefficient for men in female-dominated occupations is also negative, although not significant at the conventional level (p = 0.143; see Fig 2 and S2 Table). At first glance, the differences between women and men in female-dominated occupations might be puzzling. However, when examining occupational prestige scores in female-dominated occupations (results not shown), we find that men in the sample on average work in positions that score six points higher on the occupational prestige scale than women. Notwithstanding the control of ISEI scores in the analysis, higher occupational positions might be associated with other traits that attenuate the negative relationship between female-dominated occupations and WFH for men. Further, we find that for both women and men, a high occupational score is associated with a higher likelihood of working from home. Every 10-point increase in the occupational prestige is positively associated with the likelihood of working from home by eight percentage points; men are ten percentage points and women seven percentage points more likely to work from home with every 10-point increase on the occupational prestige scale.

Regarding *family demands*, performing all or most of the household chores is largely not associated with the likelihood of working from home during the pandemic. However, results show that men are 14 percentage points more likely to work from home when they have the main responsibility for childcare, while the association for women is much smaller and not significant (see Fig 2 and S2 Table). Interestingly, identically to prior to the pandemic [4], men with children seem to have more flexibility in negotiating working from home compared to women. However, as shown in Fig 1C, there are only 10 men in our sample that report doing most or all the care work. Also, this coefficient is not significant at the conventional level (p = 0.071); we thus hesitate to assign too much meaning to this result.

With respect to *education*, we find that highly educated men and women are significantly more likely to have transitioned to working from home during the pandemic. Compared to respondents with intermediate education, women are 25 percentage points more likely to have worked from home if they are highly educated, and men 15 percentage points.

Regarding the control variables, we find that lock-down regulations still in place are associated with higher WFH. Living in rural areas, in eastern parts of Germany or having a migration background in the first or second generation reduces the likelihood of working from home.

**Fig 3:**
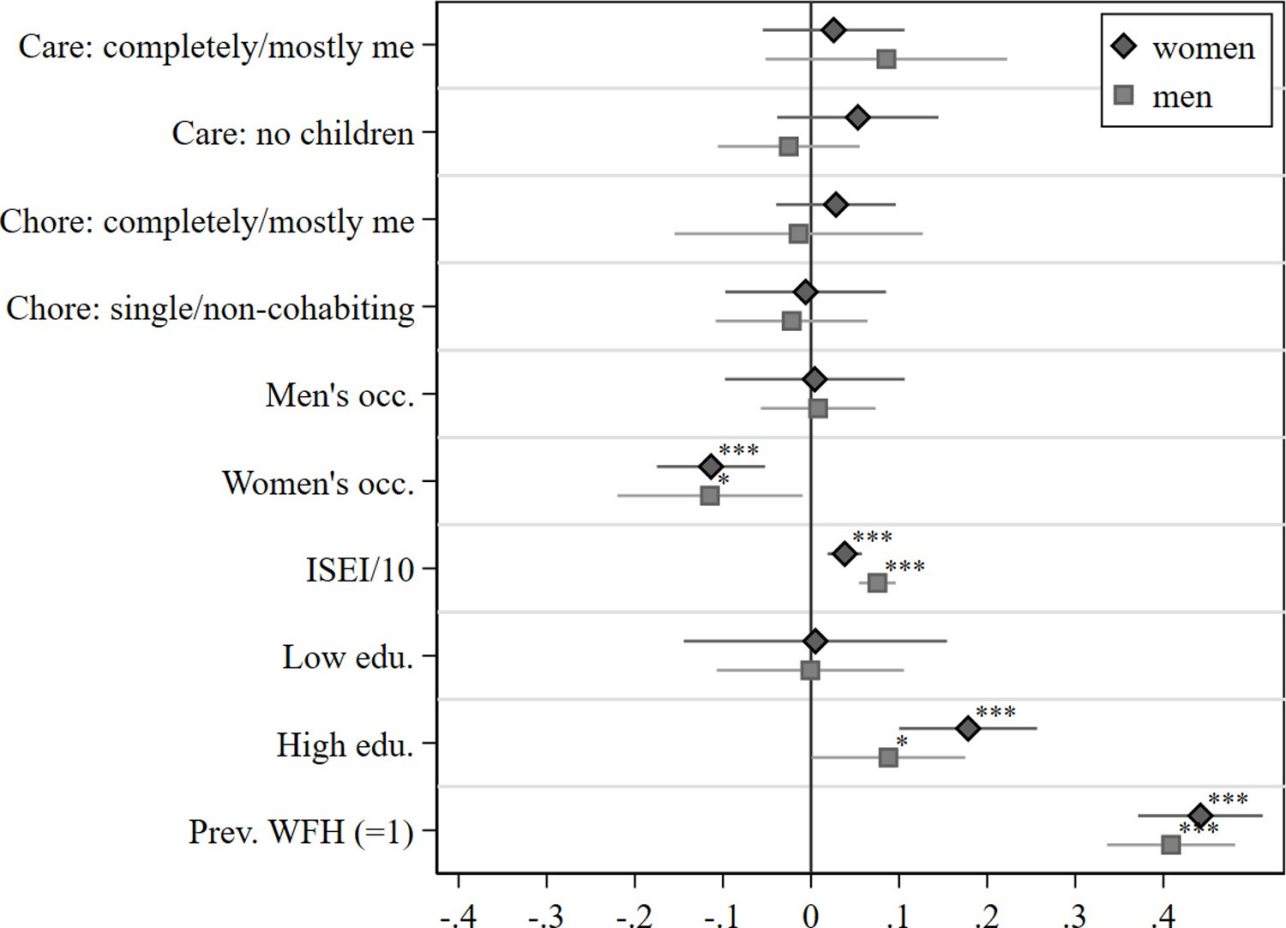
Work from home regressed on family responsibilities, occupational characteristics, education, and WFH prior to COVID-19. Note: Plot of Linear Probability Model coefficients (see S3 Table for the full models). Based on *pairfam*-COVID-19 survey and *pairfam*, release 12.0, and a special evaluation of the German LFS 2019, not weighted. Reference groups: Care: split even/mostly partner/completely partner; Chore: split even/mostly partner/completely partner; mixed occupation; intermediate education. Controls (not shown): enrolled in education, East, migration background, rural, cohorts. ^+^ *p* < 0.10 * *p* < 0.05, ** *p* < 0.01, *** *p* < 0.001.

As noted earlier, respondents in the COVID-19 study were asked whether their working arrangements changed due to the pandemic and were only then queried regarding their working-from-home circumstances. However, a majority (55%) of relatively few respondents in the sample who reported working from home at least partially prior to the corona crisis, also stated that they had a change in working arrangements to working from home during the pandemic. It is reasonable to assume that they probably worked from home more frequently than they had before COVID-19. By flagging respondents who reported having worked from home prior to the outbreak of the pandemic, we added this information to the further model specification. Results are depicted in Fig 3, as well as in S3 Table, and are very similar to the previous findings. Again, education, occupational prestige, and working in a female-dominated occupation are important predictors for working from home. As expected, having had worked from home prior to the pandemic is the most powerful predictor. Women who worked from home prior to COVID-19 are 44 percentage points more likely to do so during the pandemic, while the respective coefficient for men amounts to 41 percentage points. However, the coefficients in Fig 3 now represent the associations of WFH and the model’s covariates purged of WFH experience prior to the pandemic. In terms of statistical consequences, it is important to note that many respondents who worked from home before the pandemic also did so during the pandemic, i.e. the error terms between our dependent variable and one of our independent variables are highly correlated, which violates the model assumptions; thus, we should be careful not to overstate the statistical value of this model.

### Sensitivity analysis

In order to assess the robustness of our results we constructed four tests.

First, we re-ran our model adding the occupational position (white- and blue-collar worker, civil servant, self-employed, and being enrolled or in training) as an explanatory variable (S4 Table). Results are similar to the previous model specifications and in addition, they underscore the importance of occupational position for WFH. We find that civil servants and white-collar employees have jobs that allow incumbents to work from home, over and above the educational level and occupational prestige. Furthermore, men have a higher likelihood of WFH if they are self-employed, while for women this is not the case. It seems that gendered occupational segregation is also apparent in self-employment, with women concentrated in businesses ill-suited for WFH. Still, since the self-employed might have more schedule and workplace control or otherwise differ from regular employees, we estimated a model in which we excluded the self-employed (see S5 Table). However, we find that results largely resemble the ones presented in the main model S2 Table. Please note that our sample contains only 97 self-employed respondents.

Third, we constructed a test for our model specification due to a wide-spread belief that linear regression should not be used when the dependent variable is dichotomous. We test whether the logistic model fits the data better than the linear model and present the results as average marginal effects in S6 Table. The conclusions based on this model are the same and results are almost identical in size compared to our main linear probability model, as they should be.

Next, we ran an additional regression in which we included only respondents with children in the household. S7 Table shows that results are similar to our main model; however, if women report doing all or most of the household chores they are 11 percentage points more likely to work from home. This result, coupled with the results from S2 Table which indicate that men are more likely to work from home if they take on most or all the child care, suggests that care and household chores might not be factors that are entirely dismissive in light of WFH but nevertheless appear to be much less important than occupational characteristics. Please also note that sample sizes in S7 Table are rather small.

## Summary and discussion

With an ever-evolving global pandemic, working from home is one of the key mechanisms in maintaining control of COVID-19. This concern is even more significant since recent research suggests that the workplace is a critical setting for being exposed to and contracting COVID-19 [18]. In addition, working from home is often identified as one of the key methods for reconciling work and family commitments. Due to school closures, the pandemic has increased family responsibilities in childcare and the amount of domestic work performed at home. The significance of WFH may be reduced but it will not vanish when COVID-19 regulations are over. Thus, WFH under the pandemic conditions can be seen as a test of the overall capacity for the spread of WFH. Legal obligations to work from home imposed during the pandemic obviously make it harder for employers to apply their own criteria for WFH or discriminate among employees when granting WFH. Rather, we assume that the pandemic revealed the limits to WFH and is thus a perfect set-up to re-evaluate and juxtapose the importance of occupations/job characteristics on the one hand and family/household arrangements on the other for the propensity of working from home. Unlike many studies that emerged after the outbreak of COVID-19, and which were based on convenience samples, we can address these questions using high-quality nation-wide household panel survey data that are particularly suitable to address crucial questions about the individual and societal consequences of the pandemic. We can examine individuals in their household constellations, as well as from a longitudinal perspective.

Our results show that occupational traits, especially the gender composition of an occupation, are an important predictor for working from home. Women in female-dominated occupations are less often in a position to work from home. Occupational segregation, defined as the tendency of men and women to work in different occupations, thus plays an important role not only in well-known phenomena such as gendered wage differences, but is also associated with WFH and, ultimately, the ability to protect personal health during a pandemic. This is a novel finding which has not been examined in research on WFH, neither before nor during the pandemic.

Further, our study corroborates the well-known findings that it is especially the highly educated, as well as people who work in high-prestige occupations, who have the opportunity to work from home. Family configurations and care obligations do not seem to be decisive for WFH. Neither primary responsibility for the household nor for care work are significantly associated with the likelihood of working from home, in spite of the unprecedented situation of school and daycare closure and increased parental responsibilities for children’s (early) education. To be precise, we detected some evidence suggesting that family responsibilities have played a minor role with regard to the distribution of WFH, such as fathers solely or mainly responsible for childcare working from home. However, these results rest on a very small sample size and are not reliable. Larger data sets that contain more couples and parents in paid work are required for testing this association.

In general, a major drawback of our study is that we cannot claim causality. Different analytical techniques which would enable one to draw causal conclusions are not possible with our data because the content as well as wording of specific items differ between regular *pairfam* waves and the here employed COVID-19 survey. This poses several challenges. We do not know, for example, whether respondents work from home because they have care responsibilities and they deliberately sought WFH or they were sent to “homeoffice” during the lockdown and as a consequence they took over care responsibilities and household chores. This might not be a major drawback in our study since we only found tentative evidence that family responsibilities might play a minor role in WFH, but this is certainly something that should be re-evaluated with a larger survey. Related to this are restrictions we experienced with small sample sizes. It would be intriguing to examine in detail intra-couple distribution of care and housework and WFH of both partners; however, sample sizes in our study are too small for such an endeavor.

Overall, our results demonstrate that working from home is feasible for a substantial proportion of employees. Should the legal rights in this respect be expanded beyond the COVID-19 pandemic, this will certainly provide welcome flexibility for some employees. However, policymakers should be aware of the limitations of such measures and should not proclaim them to be a solution that promotes the reconciliation of work and family responsibilities, and particularly not as an answer to gender inequalities at home and in the labor market. Employees in female-dominated occupations, who are mostly women by definition, are significantly less likely to be offered the opportunity to work from home, even in the extreme situation of a pandemic. Women who could disproportionately profit from legal rights to work from home are, first and foremost, highly educated women in white collar and civil servant jobs.

## Supporting information

S1 Dataset Federal statistical office: Occupation-specific gender composition based on the German LFS 2019. (XLS)

S2 Table Estimates of the likelihood of WFH. Linear probability model.
(PDF)

S3 Table Estimates of the likelihood of WFH, including WFH prior to COVID-19. Linear probability model.
(PDF)

S4 Table Estimates of the likelihood of WFH, including occupational status. Linear probability model.
(PDF)

S5 Table Estimates of the likelihood of WFH, excluding the self-employed. Linear probability model.
(PDF)

S6 Table Estimates of the likelihood of WFH. Logistic regression, average marginal effects.
(PDF)

S7 Table Estimates of the likelihood of WFH, only respondents with children. Linear probability model.
(PDF)

S1 Materials Stata syntax. (ZIP)
