# Supplementary material for: The significance of occupations, family responsibilities, and gender for working from home: Lessons from COVID-19
Source: PLoS One. 2022 Jun 13;17(6):e0266393. doi: 10.1371/journal.pone.0266393 (PMC9191736; doi:10.1371/journal.pone.0266393)
Supplement: S2 Table — Linear probability model. (PDF) [file pone.0266393.s002.pdf]

S1 Table. Estimates of the likelihood of WFH. Linear Probability Model.

	Total		Women		Men		Δ(women-men)	
Women (=1)	-0.00	(0.03)	X		X			
Family responsibilities								
Care: even/mostly partner/other					Ref.			
Care: completely/mostly me	0.03	(0.04)	0.04	(0.05)	0.14^{+}	(0.08)	-0.10	(0.09)
Care: no children	0.00	(0.03)	0.06	(0.05)	-0.03	(0.04)	0.09	(0.07)
Chore: even/mostly partner/other					Ref.			
Chore: completely/mostly me	0.02	(0.03)	0.06	(0.04)	-0.02	(0.08)	0.08	(0.08)
Chore: single/non-cohabiting	-0.02	(0.03)	0.01	(0.05)	-0.02	(0.05)	0.03	(0.07)
Occupational characteristics								
Mixed occupation					Ref.			
Men's occupation	0.03	(0.03)	0.06	(0.05)	0.02	(0.04)	0.04	(0.07)
Women's occupation	-0.13***	(0.03)	-0.13***	(0.03)	-0.08	(0.05)	-0.05	(0.06)
ISEI/10	0.08***	(0.01)	0.07***	(0.01)	0.10***	(0.01)	-0.03*	(0.02)
Education								
Intermediate education					Ref.			
Low education	-0.02	(0.05)	-0.03	(0.08)	-0.03	(0.06)	0.00	(0.10)
High education	0.21***	(0.03)	0.25***	(0.04)	0.15**	(0.05)	0.11	(0.07)
Enrolled	0.16*	(0.08)	0.08	(0.11)	0.22*	(0.11)	-0.14	(0.16)
Controls								
Lock-down in place (=1)	0.08*	(0.03)	0.06	(0.04)	0.11*	(0.05)	-0.05	(0.07)
East (=1)	-0.06*	(0.03)	-0.04	(0.04)	-0.08*	(0.04)	0.05	(0.06)
Migration background (=1)	-0.06^{+}	(0.03)	-0.10*	(0.04)	0.01	(0.05)	-0.10	(0.07)
Rural (=1)	-0.08**	(0.03)	-0.06	(0.04)	-0.11*	(0.04)	0.05	(0.06)
Cohorts	✓		✓		✓		✓	
Constant	-0.03	(0.07)	-0.00	(0.10)	-0.12	(0.09)		
Observations	1414		773		641			
R^2	0.29		0.27		0.33			

Note: Based on *pairfam*-COVID-19 survey, *pairfam*, release 12.0, and a special evaluation of the German LFS 2019, own calculations, not weighted; standard errors in parentheses. $^{+}$ $p < 0.10$ * $p < 0.05$, ** $p < 0.01$, *** $p < 0.001$.
