# Supplementary material for: The significance of occupations, family responsibilities, and gender for working from home: Lessons from COVID-19
Source: PLoS One. 2022 Jun 13;17(6):e0266393. doi: 10.1371/journal.pone.0266393 (PMC9191736; doi:10.1371/journal.pone.0266393)
Supplement: S3 Table — Linear probability model. (PDF) [file pone.0266393.s003.pdf]

S2 Table. Estimates of the likelihood of WFH, including WFH prior to COVID-19. Linear Probability Model.

	Total		Women		Men	
Women (=1)	0.00	(0.03)	X		X	
Family responsibilities						
Care: even/mostly partner/other			Ref.			
Care: completely/mostly me	0.01	(0.03)	0.03	(0.04)	0.09	(0.07)
Care: no children	0.00	(0.03)	0.05	(0.05)	-0.03	(0.04)
Chore: even/mostly partner/other			Ref.			
Chore: completely/mostly me	0.00	(0.03)	0.03	(0.03)	-0.01	(0.07)
Chore: single/non-cohabiting	-0.02	(0.03)	-0.01	(0.05)	-0.02	(0.04)
Occupational characteristics						
Mixed occupation			Ref.			
Men's occupation	0.00	(0.03)	0.00	(0.05)	0.01	(0.03)
Women's occupation	-0.12^{***}	(0.03)	-0.11^{***}	(0.03)	-0.11^{*}	(0.05)
ISEI/10	0.06^{***}	(0.01)	0.04^{***}	(0.01)	0.08^{***}	(0.01)
Education						
Intermediate education			Ref.			
Low education	0.01	(0.04)	0.00	(0.08)	-0.00	(0.05)
High education	0.14^{***}	(0.03)	0.18^{***}	(0.04)	0.09^{*}	(0.04)
Enrolled	0.14	(0.09)	0.09	(0.13)	0.16	(0.12)
Controls						
Lock-down in place (=1)	0.05^{+}	(0.03)	0.05	(0.04)	0.07	(0.05)
East (=1)	-0.02	(0.03)	-0.00	(0.04)	-0.04	(0.04)
Migration background (=1)	-0.03	(0.03)	-0.07^{+}	(0.04)	0.04	(0.05)
Rural (=1)	-0.06^{*}	(0.03)	-0.04	(0.04)	-0.09^{*}	(0.04)
Pre-COVID WFH (=1)	0.43^{***}	(0.03)	0.44^{***}	(0.04)	0.41^{***}	(0.04)
Cohorts	✓		✓		✓	
Constant	0.02	(0.06)	0.06	(0.09)	-0.06	(0.08)
Observations	1382		753		629	
R^2	0.42		0.40		0.45	

Note: Based on *pairfam*-COVID-19 survey and *pairfam*, release 12.0, and a special evaluation of the German LFS 2019, own calculations, not weighted; standard errors in parentheses. $^{+}$ $p < 0.10$ * $p < 0.05$, ** $p < 0.01$, *** $p < 0.001$.
