# Supplementary material for: The significance of occupations, family responsibilities, and gender for working from home: Lessons from COVID-19
Source: PLoS One. 2022 Jun 13;17(6):e0266393. doi: 10.1371/journal.pone.0266393 (PMC9191736; doi:10.1371/journal.pone.0266393)
Supplement: S4 Table — Linear probability model. (PDF) [file pone.0266393.s004.pdf]

S3 Table. Estimates of the likelihood of WFH, including occupational status. Linear Probability Model.

	Total		Women		Men	
Women (=1)	-0.00	(0.03)				
Family responsibilities						
Care: even/mostly partner/other			Ref.			
Care: completely/mostly me	0.04	(0.04)	0.05	(0.05)	0.14^{+}	(0.08)
Care: no children	0.01	(0.03)	0.07	(0.05)	-0.03	(0.05)
Chore: even/mostly partner/other			Ref.			
Chore: completely/mostly me	0.02	(0.03)	0.05	(0.04)	-0.03	(0.08)
Chore: single/non-cohabiting	-0.01	(0.03)	0.01	(0.05)	-0.01	(0.05)
Occupational characteristics						
Mixed occupation			Ref.			
Men's occupation	0.05	(0.03)	0.08	(0.05)	0.04	(0.04)
Women's occupation	-0.15^{***}	(0.03)	-0.16^{***}	(0.03)	-0.09	(0.06)
ISEI/10	0.07^{***}	(0.01)	0.05^{***}	(0.01)	0.08^{***}	(0.01)
Blue-collar			Ref.			
White-collar	0.21^{***}	(0.04)	0.17^{**}	(0.07)	0.23^{***}	(0.05)
Civil servants	0.34^{***}	(0.06)	0.39^{***}	(0.08)	0.24^{**}	(0.08)
Self-employed	0.08	(0.07)	-0.07	(0.10)	0.21^{*}	(0.09)
Enrolled/in training	0.24^{*}	(0.10)	0.41^{**}	(0.15)	0.06	(0.14)
Education						
Intermediate education			Ref.			
Low education	-0.01	(0.05)	-0.02	(0.09)	-0.01	(0.06)
High education	0.20^{***}	(0.03)	0.24^{***}	(0.04)	0.13^{**}	(0.05)
Enrolled	0.16^{+}	(0.08)	0.09	(0.12)	0.22^{+}	(0.12)
Controls						
Lock-down in place (=1)	0.08^{*}	(0.03)	0.06	(0.05)	0.11^{*}	(0.06)
East (=1)	-0.04	(0.03)	-0.01	(0.04)	-0.08	(0.05)
Migration background (=1)	-0.05	(0.03)	-0.08^{+}	(0.04)	0.00	(0.05)
Rural (=1)	-0.08^{**}	(0.03)	-0.05	(0.04)	-0.10^{*}	(0.05)
Cohorts	✓		✓		✓	
Constant	-0.15^{*}	(0.07)	-0.12	(0.11)	-0.22^{*}	(0.09)
Observations	1353		746		607	
R^2	0.30		0.31		0.34	

Note: Based on *pairfam*-COVID-19 survey and *pairfam*, release 12.0, and a special evaluation of the German LFS 2019, own calculations, not weighted; standard errors in parentheses. $^{+}$ $p < 0.10$ * $p < 0.05$, ** $p < 0.01$, *** $p < 0.001$.
