# Supplementary material for: The significance of occupations, family responsibilities, and gender for working from home: Lessons from COVID-19
Source: PLoS One. 2022 Jun 13;17(6):e0266393. doi: 10.1371/journal.pone.0266393 (PMC9191736; doi:10.1371/journal.pone.0266393)
Supplement: S6 Table — Logistic regression, average marginal effects. (PDF) [file pone.0266393.s006.pdf]

S5 Table. Estimates of the likelihood of WFH. Logistic regression, average marginal effects.

Women (=1)	0.00	(0.03)
Family responsibilities		
Care: even/mostly partner/other	Ref.	
Care: completely/mostly me	0.03	(0.04)
Care: no children	-0.00	(0.03)
Chore: even/mostly partner/other	Ref.	
Chore: completely/mostly me	0.02	(0.03)
Chore: single/non-cohabiting	-0.02	(0.03)
Occupational characteristics		
Mixed occupation	Ref.	
Men's occupation	0.02	(0.03)
Women's occupation	-0.12***	(0.03)
ISEI/10	0.07***	(0.01)
Education		
Intermediate education	Ref.	
Low education	-0.03	(0.06)
High education	0.19***	(0.03)
Enrolled	0.19*	(0.10)
Controls		
Lock-down in place (=1)	0.08*	(0.03)
East (=1)	-0.06*	(0.03)
Migration background (=1)	-0.07*	(0.03)
Rural (=1)	-0.08**	(0.03)
Cohorts	✓	
Observations	1414	
McFadden R^2	0.23	

Note: Based on *pairfam*-COVID-19 survey and *pairfam*, release 12.0, and a special evaluation of the German LFS 2019, own calculations, not weighted; standard errors in parentheses. $^{+}$ $p < 0.10$ * $p < 0.05$, ** $p < 0.01$, *** $p < 0.001$.
