# Supplementary material for: The significance of occupations, family responsibilities, and gender for working from home: Lessons from COVID-19
Source: PLoS One. 2022 Jun 13;17(6):e0266393. doi: 10.1371/journal.pone.0266393 (PMC9191736; doi:10.1371/journal.pone.0266393)
Supplement: S7 Table — Linear probability model. (PDF) [file pone.0266393.s007.pdf]

S6 Table. Estimates of the likelihood of WFH, only respondents with children. Linear Probability Model.

	Total		Women		Men	
Women (=1)	-0.13*	(0.05)	X		X	
Family responsibilities						
Care: even/mostly partner/other			Ref.			
Care: completely/mostly me	0.04	(0.04)	0.03	(0.05)	0.12	(0.09)
Chore: even/mostly partner/other			Ref.			
Chore: completely/mostly me	0.09+	(0.05)	0.11*	(0.05)	-0.03	(0.11)
Occupational characteristics						
Mixed occupation			Ref.			
Men's occupation	-0.04	(0.04)	0.00	(0.09)	-0.05	(0.05)
Women's occupation	-0.12**	(0.04)	-0.13**	(0.05)	-0.11	(0.07)
ISEI/10	0.07***	(0.01)	0.05**	(0.02)	0.10***	(0.02)
Education						
Intermediate education			Ref.			
Low education	-0.06	(0.07)	-0.11	(0.10)	-0.07	(0.12)
High education	0.21***	(0.05)	0.28***	(0.06)	0.09	(0.08)
Controls						
Lock-down in place (=1)	0.13*	(0.05)	0.05	(0.07)	0.27**	(0.08)
East (=1)	-0.08*	(0.04)	-0.03	(0.05)	-0.14**	(0.05)
Migration background (=1)	-0.06	(0.05)	-0.11	(0.06)	0.03	(0.08)
Rural (=1)	-0.15***	(0.04)	-0.09+	(0.05)	-0.21**	(0.06)
Cohorts	✓		✓		✓	
Constant	0.03	(0.11)	-0.12	(0.16)	-0.03	(0.16)
Observations	663		377		286	
R^2	0.30		0.28		0.37	

Note: Based on *pairfam*-COVID-19 survey and *pairfam*, release 12.0, and a special evaluation of the German LFS 2019, own calculations, not weighted; standard errors in parentheses. $^{+}$ $p < 0.10$ * $p < 0.05$, ** $p < 0.01$, *** $p < 0.001$.
